# Use of Dynamic Spinal Instruments (Dynesys) in Adult Spinal Deformities According to Silva–Lenke and Berjano–Lamartina Classifications

**DOI:** 10.3390/diagnostics14050549

**Published:** 2024-03-05

**Authors:** Mehmet Yigit Akgun, Ege Anil Ucar, Cemil Cihad Gedik, Caner Gunerbuyuk, Mehdi Hekimoglu, Onder Cerezci, Tunc Oktenoglu, Mehdi Sasani, Ozkan Ates, Ali Fahir Ozer

**Affiliations:** 1 Department of Neurosurgery, Koc University Hospital, 34010 Istanbul, Turkey; myigitakgun@gmail.com (M.Y.A.); tuncoktenoglu@gmail.com (T.O.); sasanim@gmail.com (M.S.); atesozkan@hotmail.com (O.A.); 2 Spine Center, Koc University Hospital, 34010 Istanbul, Turkey; drcaner@yahoo.com; 3 Medical Faculty, Koc Univesity School of Medicine, 34010 Istanbul, Turkey; eucar17@ku.edu.tr; 4 Department of Orthopaedics and Traumatology, Koc University Hospital, 34010 Istanbul, Turkey; cgedikkuh@gmail.com; 5 Department of Neurosurgery, American Hospital, 34010 Istanbul, Turkey; mehdih@amerikanhastanesi.org; 6 Department of Physical Medicine and Rehabilitation, American Hospital, 34010 Istanbul, Turkey; onder@amerikanhastanesi.org

**Keywords:** Dynesys system, spinal deformity classification, dynamic stabilization

## Abstract

Background: Adult spinal deformities (ASD) present complex challenges in spine surgery. The diverse nature of these deformities requires a comprehensive understanding of their classification and treatment options. Traditional approaches, such as fusion and rigid stabilization are associated with complications, including screw loosening, breakage, proximal junctional kyphosis (PJK), and pseudoarthrosis. Dynamic stabilization techniques have emerged as promising alternatives, to reduce these complications and preserve spinal motion. Objective: This study investigated the effectiveness of dynamic stabilization using the Dynesys system in the surgical treatment of adult degenerative spinal deformities, with a particular emphasis on their classification. Methods: ASDs were classified according to the Berjano–Lamartina (BL) and Silva–Lenke (SL) classifications. We analyzed the efficacy of the Dynesys system in enhancing sagittal balance, radiological parameters, and clinical outcomes in this context. Results: Dynamic stabilization of patients with ASDs using the Dynesys system significantly improved the visual analog scale and Oswestry Disability Index scores and decreased the complication rates. Patients with BL types 2, 3, and 4 experienced a significant improvement in sagittal balance followed by sagittal vertical axis measurements (*p* = 0.045, *p* = 0.015, and *p* < 0.0001, respectively). Conclusion: The SL and BL classifications, which were originally developed for rigid spinal stabilization, can be applied in dynamic stabilization. Furthermore, dynamic stabilization using the Dynesys system can be used as an alternative to rigid stabilization in SL levels 2 and 3, and BL types 1, 2, and 3, and in some patients with type 4 ASDs.

## 1. Introduction

Adult spinal deformities (ASDs) are one of the most challenging issues in spine surgery and are characterized by their intricate etiopathogenesis, multifaceted classification, and diverse clinical presentations [1,2,3]. These conditions, broadly categorized as sagittal, coronal, and axial plane deformities, require individualized treatment strategies.

ASDs are quite common in individuals aged > 65 years, affecting a substantial portion of this demographic population, with prevalence rates ranging from 32% to 68% [4,5]. Such a high incidence of spinal deformities among elderly individuals, a demographic population often burdened by comorbidities, underscores their clinical importance.

The treatment method for each deformity is different. Moreover, most patients have more than one deformity as well as a multisegmental pathology. For instance, a patient with degenerative lumbar scoliosis may have both rotational and kyphotic deformities. This complex clinical picture is further compounded by the emergence of chronic instability over time. Hence, deformity surgery is one of the most challenging issues of spine surgery because all these factors affect each other.

As seen in Ref. [6], patients with degenerative deformities often have comorbidities such as hyper-tension, diabetes, and respiratory diseases [7,8]. Consequently, treatment is aimed at alleviating symptoms and preventing further instability rather than solely focusing on deformity correction. The only method indisputably used for treating all types of deformity surgery is instrumentation and fusion surgery. Although simple spinal decompression can alleviate radicular pain in the lower limbs, it often results in poor long-term outcomes, which are closely related to deformity progression.

Recognizing the limitations and complications associated with fusion procedures has led to advances in motion preservation strategies. In recent years, spinal surgery has witnessed remarkable advancements in posterior dynamic stabilization techniques [9], which aim to preserve spinal motion while effectively addressing degenerative spinal deformities. Dynamic stabilization systems in spine surgery represent a paradigm shift from traditional fusion approaches, providing an innovative solution for the treatment of ASDs. Unlike fusion, which aims to immobilize and fuse affected segments, dynamic stabilization systems maintain spinal mobility while providing stability. A prominent example is the Dynesys system, which is a posterior dynamic stabilization technique that has garnered attention for its potential benefits. It is a nonfusion pedicle screw stabilization system designed to preserve a degree of natural vertebral motion and can be adjusted by the surgeon. Complete rigid fixation and prevention of all motions within fused segments predisposes the materials to higher stress and the spine to accelerated degenerative changes in the adjacent segments [10] and affects the overall functioning in elderly patients. In this regard, dynamic stabilization using the Dynesys system is a promising method for improving patient outcomes and addressing the complications associated with ASDs, providing greater physiological stabilization than traditional alternatives.

The Silva–Lenke [11] (SL) and Berjano–Lamartina [12] (BL) classification systems are well established within the field of ASDs. These classification systems were originally de-vised for categorizing deformities in the context of rigid stabilization procedures and have played a pivotal role in providing the structure and organization for spinal surgeons and guiding the treatment strategy. Nevertheless, as spine surgery has evolved and dynamic stabilization techniques have gained prominence, the question arises whether these classification systems can be effectively repurposed to evaluate and guide dynamic stabilization procedures.

We conducted this study to investigate the effectiveness of the dynamic stabilization system in treating patients with ASDs and the applicability of the SL and BL classification systems in the context of dynamic stabilization of patients with ASDs using the Dynesys system.

## 2. Materials and Methods

This retrospective study was authorized by the Institutional Review Board of Koc University (protocol code 2022.021.IRB.016 and date of approval 13 January 2022) and was performed according to the principles of the Declaration of Helsinki. Informed consent was obtained from all participants included in the study.

Adult degenerative deformity cases that were operated between 2018 and 2021 were retrospectively analyzed. Patients with complete radiological and clinical follow-up/treatment, no improvement after conservative treatment, no previous lumbar fusion or stabilization surgery, and with at least 2 years of clinical and radiological follow-up were analyzed.

Patients with mobile deformities were selected as ideal candidates for dynamic stabilization surgery. In patients with kyphotic deformities, the posture in which the patient can stand upright without support and the forward-leaning posture of the spine after walking or standing were evaluated using standing lateral radiographs of the spine.

Scoliotic deformities were evaluated using anteroposterior lying and standing full-frontal and lateral radiographs and lateral bending-view radiographs. This method can help confirm mobile deformities. All spinal radiographs were obtained in this manner. Sagittal balance was evaluated with both pelvic parameters [13] (Table 1) and the sagittal vertical axis (SVA) using the C7 plumb line, i.e., measuring the distance between a vertical line drawn from the center of the C7 vertebral body and the superior–posterior endplate of the S1.

Patients were categorized according to the SL and BL classification systems. In the SL classification system, the following treatment strategies were recommended: “I, decompression alone; II, decompression and limited instrumented posterior fusion; III, decompression and lumbar curve instrumented fusion; and IV, decompression with anterior and posterior spinal fusion.” Furthermore, levels V and VI require more extended fusion and osteotomies [11]. For patients categorized under the BL classification system, if a mobile deformity was observed in certain cases of type I, type II, and type III, as well as in type IVa and type IVb patients, it might be feasible to consider dynamic stabilization as a stand-alone treatment method [12]. Standing full-frontal and lateral radiographs were used by two senior neurosurgeons to determine the SL and BL classifications of each patient. The recommended treatment scheme was applied as dynamic stabilization using the Dynesys system for each group of our patients. Hence, our aim was to create treatment schemes according to common language and classifications.

Considering the etiology of the patients, the spinal deformities in our cohort developed because of the degenerative process. All patients underwent dual-energy X-ray absorptiometry, magnetic resonance imaging, and computed tomography. The presence of scoliosis, kyphosis, and kyphoscoliosis was examined in each patient, and the preoperative values were recorded (Table 1). Kyphotic and scoliotic Cobb angles were evaluated by measuring the angle formed between two lines drawn from the respective endplates of the deformity.

A neuromonitoring system was used in all patients. In cases with cord or root compression and neurological deficits, decompression at the required levels was performed primarily. Furthermore, osteotomies were performed at the required levels according to the preoperative planning. The transpedicular screw system was placed using the Wiltse method [14]. The Dynesys system [15] was chosen as the dynamic stabilization system. In patients with a scoliotic deformity, because the deformity is partially corrected in the prone position, the remaining deformity is corrected as much as possible by cutting the spacers shorter than normal and providing greater torque than normal in the concave part of the deformity. In the case of kyphotic deformities, normal sagittal balance is achieved by positioning the table, accompanied by fluoroscopy, and the rods are placed at this position. In this manner, the impaired sagittal balance is restored to normal. Because several patients have both deformities concomitantly, both procedures are performed simultaneously.

All patients were evaluated using the visual analog scale (VAS) and Oswestry Disability Index (ODI) scores in the preoperative period, in the early postoperative period, and at the 12- and 24-month postoperative follow-up. The operating time, blood loss, and complications were also documented.

### Statistical Analyses

Statistical analyses were conducted using the SPSS software (Statistical Package for the Social Sciences; SPSS Inc., Chicago, IL, USA) version 22 and GraphPad (Prism) version 9. Continuous variables were presented as mean ± standard deviation. For repeated and two measurements, the paired samples *t*-test was used. ANOVA was used for more than two measurements. The level of statistical significance was set at *p* < 0.05 for all analyses. *p* values <0.05, <0.01, <0.001, and <0.0001 were denoted by “*”, “**”, “***”, and “****”, respectively.

## 3. Results

A total of 68 patients, including 28 men and 40 women, were included in this study. The patients’ mean age was 62.18 ± 15.42 (range: 49–85) years. The mean follow-up period was 39.12 (range: 24–120) months. The Dynesys system was used in all patients (Table 2).

**Figure 1:**
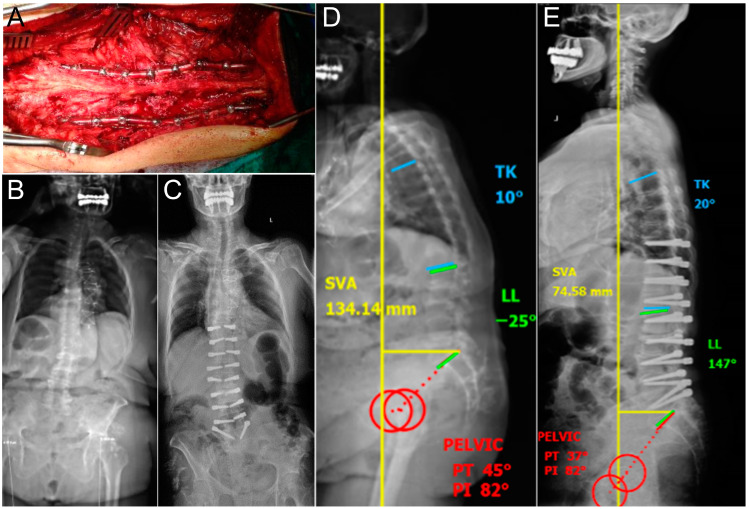
Treatment of kyphosis with Dynesys dynamic stabilization. (**A**) Intraoperative image showing the posterior stabilization with Dynesys, (**B**) pre-operative and (**C**) post-operative coronal dynamic X-rays. (**D**) Pre-operative and (**E**) post-operative dynamic sagittal X-rays displaying sagittal parameters of the spine.

Patients were divided into groups according to the BL and SL classification systems. The preoperative and postoperative pelvic parameters, including sacral slope (SS), pelvic tilt (PT), and pelvic incidence, are presented in Table 1. Significant reductions in scoliotic Cobb angles were observed within each SL and BL group (Table 3), especially in the LS level 3 patients, from a mean of 34.50° to 16.50° (*p* < 0.001). Interestingly, the SVA remained relatively stable in patients categorized as BL type 1, whereas patients categorized as BL types 2, 3, and 4 experienced substantial postoperative SVA reductions (*p* = 0.045, *p* = 0.015, and *p* < 0.0001, respectively) (Figure 1).

We evaluated the clinical outcomes at 1- and 2-year follow-up intervals. Adequate improvement in deformity could not be achieved in four patients, all of whom were classified as BL type 4 and SL level 3. Highly satisfactory results were obtained in the remaining 64 patients (94.1%). A remarkable reduction was observed in both the VAS and ODI scores when the preoperative and postoperative measurements were compared. Specifically, the preoperative mean VAS and ODI scores were 6.88 and 68.22, respectively, showing a substantial improvement from 2.43 and 26.95, respectively, at the 6-month postoperative assessment (*p* < 0.001). However, there were exceptions in the comparison between the postoperative 12th and 24th month for both VAS and ODI scores, as detailed in Table 4.

Coronal and sagittal correction losses were detected by measuring the Cobb angle and SVA measurements on spinal X-rays obtained at a 6-month follow-up with early post-operative measurements. Nonetheless, these correction losses were not statistically significant (*p* > 0.05). Moreover, at the end of the 12- and 24-month follow-up, there was no significant progression in these radiological measurements.

**Figure 2:**
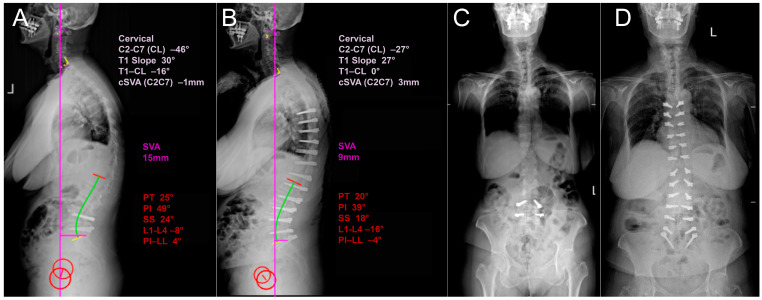
Dynamic X-rays of a patient with sagittal deformity. (**A**) Pre-operative and (**B**) post-operative sagittal dynamic X-rays displaying parameters of the spine. (**C**) Pre-operative and (**D**) post-operative dynamic coronal X-rays.

**Figure 3:**
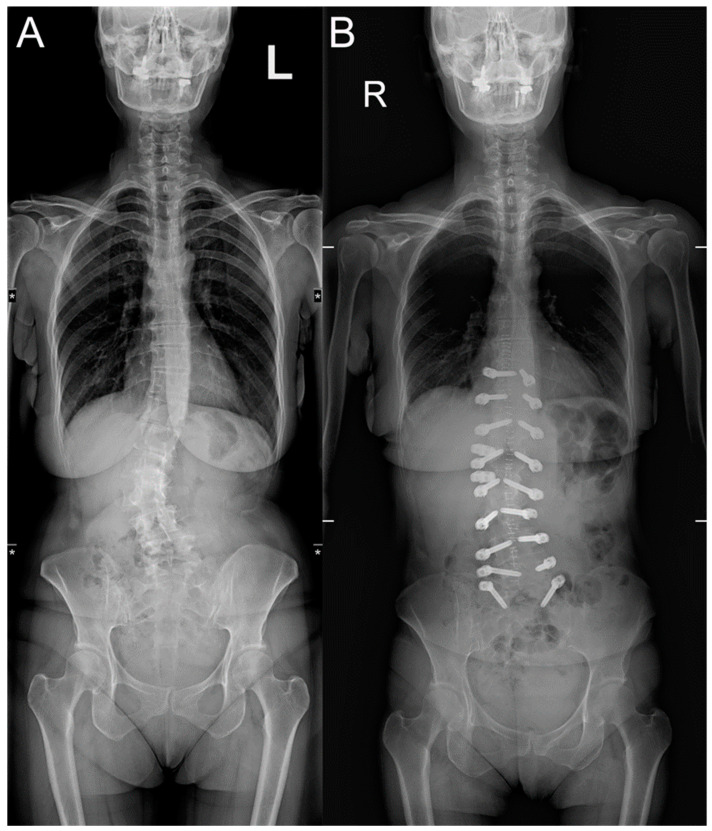
Adult scoliotic deformity treated with Dynesys dynamic stabilization. (**A**) Pre-operative and (**B**) post-operative dynamic coronal X-rays.

**Figure 4:**
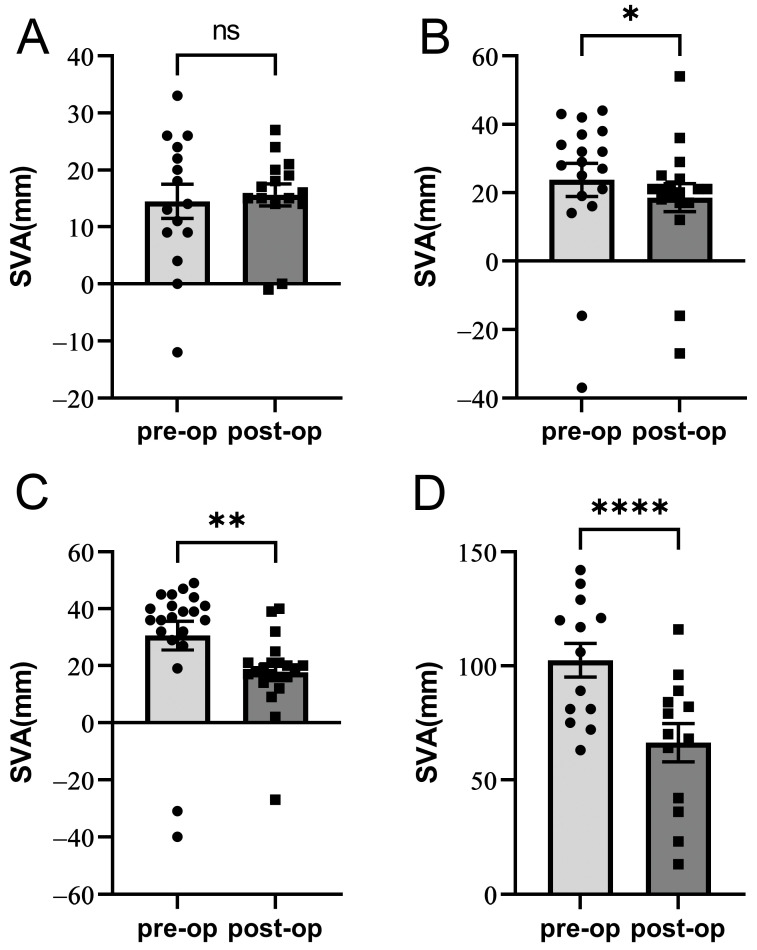
Surgical outcome of sagittal balance according to Berjano–Lamartina (BL) classification. Sagittal vertical axis is used as a parameter for sagittal balance. (**A**) BL type 1, (**B**) BL type 2, (**C**) BL type 3, and (**D**) BL type 4. “*”, “**”, and “****” denote *p* values of <0.05, <0.01, and <0.0001, respectively. ns: nonsignificant.

The mean number of fixed segments was 6.6  ±  2.9, with a mean operating time of 229.3  ±  40.6 min and an intraoperative blood loss of 611.7  ±  285.7 mL. Complications were limited to subcutaneous hematoma and superficial tissue infection. We also detected subclinical proximal junctional kyphosis (PJK) in two patients (one BL type 4 SL level 3 and one BL type 3 SL level 2) and subclinical adjacent segment disease in three patients (two BL type 3 SL level 3 and one BL type 3 SL level 2). Radiographic evidence of screw loosening was observed in three patients, but none required revision surgery due to screw malposition, adjacent segment disease, rod rupture, or screw loosening. There was no mortality in this study. Selected cases from this series are illustrated in Figure 2, Figure 3 and Figure 4.

## 4. Discussion

ASDs are complex and require a comprehensive understanding of their classification and treatment options. Traditional approaches, such as fusion and rigid stabilization, can cause complications. Dynamic stabilization techniques aim at reducing these complications and preserving spinal motion. This study classified ASDs according to the BL and SL classification systems. We analyzed the impact of the Dynesys system on sagittal balance, radiological parameters, and clinical outcomes. We also evaluated whether the BL and SL classification systems, originally developed for rigid spinal stabilization, can be applied in cases of dynamic stabilization.

When patients were categorized according to the SL classification, we found favorable outcomes of the surgery in SL levels 2 and 3 patients by dynamic stabilization. Their VAS and ODI scores and scoliotic Cobb angles significantly improved. Therefore, we recommend dynamic stabilization as an alternative to rigid stabilization in patients with ASDs categorized as SL levels 2 and 3. Although we can discuss the potential benefits of dynamic stabilization at SL level 4, it could not be added in this discussion in our cohort as there were no patients in this group.

We also focused on the BL classification system [12], in which clinical evaluations such as SL are inconspicuous and based on radiological evaluations. This classification was developed primarily to make surgical planning easier [12]. It was later developed with additional articles, and more detailed surgical recommendations were made, especially in the mobile segment. In this study, we primarily selected patients with a mobile deformity for dynamic stabilization. We evaluated the improvement in sagittal balance after dynamic stabilization within different B–L types. Our findings revealed a significant improvement in sagittal balance, particularly in the SVA, among patients classified as BL types 2, 3, and 4, whereas those categorized as BL type 1 showed no changes. The most drastic decrease was found in the dynamic stabilization of BL type 4 patients (Figure 4). The insignificant SVA reductions in the mildest BL type 1 group may be attributed to their initial SVA values being relatively close to the physiological values. Furthermore, it underscores that dynamic stabilization primarily aims to enhance patients’ clinical parameters rather than exclusively focusing on radiographic parameters. Therefore, we recommend that dynamic systems up to type I–III and some of type IV should be considered in the BL approach.

There are two major problems in rigid systems that can be reduced using dynamic stabilization. The first is screw loosening, and the second is rod breakage. Rod rupture is the most feared issue for surgeons in this system. In patients with impaired global sagittal alignment, rope-shaped rods are also responsible for pulling the trunk over the pelvis and maintaining the trunk in this position. Therefore, forward bending moments on the rod cause more loading. In our study, although rod rupture was not observed, screw loosening without clinical symptoms was detected in 4.4% of patients with ASDs. In the literature, the rates of adjacent segment disease vary significantly between 6% and 47% [16,17]. Nevertheless, the lower rate of adjacent segment disease might be attributed to the shorter time of patient follow-up in this cohort.

Dynamic stabilization is a surgery that should be performed in patients with slowly developing instability in the deformity. The results are acceptable in dynamic stabilizations applied to single-level pathologies. However, especially as the level increases, the problem of screw loosening also develops. Studies have reported that the incidence rate of PJK after fusion varies between 20% and 41% [18,19,20]. Several factors affect the incidence of PJK, such as body mass index, osteoporosis, severe fatty infiltration of paravertebral muscles, and overcorrection [21,22]. We aimed to reduce PJK complication rates through dynamic stabilization using the Dynesys system. Furthermore, to reduce PJK rates related to osteoporosis, we preferred two-stage surgery where T was lower than −1.5, as previously described in our study [23]. In our cohort, the rate of PJK was 3%, which is much lower than that reported in the literature. Interestingly, the average blood loss (611.7 mL) in our study was much higher than that reported by Hsieh et al. [24] (250 mL) and Yang et al. [25] (386 mL) but similar to that reported by Khalife et al. [26] (736 mL).

It is difficult to maintain sagittal balance in the spine, which deteriorates with age. In a study conducted in Japan, the normative values for both sagittal alignment and HRQoL scores varied according to age [27,28]. According to that study, advancing age resulted in an increase in PT and SVA and a decrease in LL and thoracic kyphosis. The authors re-ported a remarkable change in the spinopelvic sagittal alignment from the 7th to 8th decade. This natural history of sagittal spinopelvic alignment should be included in the surgical planning of patients with ASDs. It was assumed that overcorrection of ASDs would be necessary to counteract the effects of continued degeneration in the elderly population; however, over time, several studies have revealed that advanced age and surgical over-correction of SVA are independent risk factors for PJK and revision surgery [18,29,30,31]. Therefore, minimally invasive strategies such as dynamic stabilization provide a promising alternative for reducing complications while treating patients without overcorrection.

One of the most important details is that when patients with ASDs apply to the out-patient clinic, most of them present with root irritation findings due to neurogenic claudication or foraminal stenosis [6]. Mostly, ASDs are revealed by examinations. Again, a significant part of the patient’s state is that they are upright when they start walking but lean forward after a while. Irrespective of whether the global sagittal balance is impaired, this is the period when the patient has a golden opportunity. The patient can achieve global sagittal balance using the muscle compartment. It can insert the SVA into the sacrum. However, a simple dynamic stabilization surgery performed on the patient during this period will gain a lot for the patient and is ignored. In our surgeries, our aim is to improve the quality of life of elderly patients by aligning our treatment approach with their target posture for their age. In elderly individuals, attempting to create an ideal young spine through instrumentation and fusion often results in less-than-ideal outcomes, which are not uncommon [5]. It is crucial to recognize that surgery for every age group is unique and tailored to their specific needs. Furthermore, our patients are elderly with a heavy burden of comorbidities. Fusion surgery in this patient group tends to result in overcorrection, prolonged intensive care unit stays, and extended hospitalization periods, exacerbating additional complications [32,33]. In this regard, dynamic systems help patients navigate this process more comfortably, reducing these additional challenges.

The paradigm of spinal deformity treatment is to perform circumferential arthrodesis with restoration of pelvic parameters. Preserving motion and avoiding fusion may contribute to better patient quality of life by allowing for more natural movement. This can be particularly important in cases where deformity affects daily activities and functional capacity. Dynamic systems allow for a more tailored treatment approach, considering individual patient factors, the nature of the deformity, and the desired level of motion preservation. This flexibility may be considered an advantage in addressing a diverse range of spinal deformities.

Our study has several limitations. First, the potential of patient bias exists because of the selective inclusion of only patients with mobile deformity, which may restrict the generalizability of our findings. Second, our study lacks long-term follow-up data, and we present our results as preliminary because of the absence of minimum 5-year follow-up data, which is standard in the literature. Moreover, our cohort size is insufficient, which can affect the statistical power and precision of our analyses. Finally, our study has a retrospective design, which necessitates caution in interpretation.

This article asserts that these classifications can be effectively applied, as evidenced by positive results in the initial case series. We are currently in the process of preparing the findings from our study, using deformity cases subjected to rigid correction as the control group.

Future studies should investigate the broader applications of dynamic stabilization systems in ASD management, particularly in diverse patient cohorts with varying deformity characteristics and comorbidities. It is crucial to explore the long-term outcomes and efficacy of dynamic stabilization in addressing rigid deformities and age-specific considerations. In addition, it is necessary to evaluate the effectiveness of different dynamic stabilization techniques in patients with ASDs and their complication profiles. Larger scale prospective studies, overcoming the limitations of the present study, are required to establish the generalizability and long-term effectiveness of dynamic stabilization approaches, including the Dynesys system, in patients with ASDs. This will contribute to the advancement of surgical strategies, tailoring interventions to specific patient profiles, and optimizing outcomes for individuals with spinal deformities.

## 5. Conclusions

Dynamic systems in ASD surgery provide a compelling solution for reducing morbidity and complication rates. Early intervention during the mobile deformity phase is crucial, and our study emphasizes the effectiveness of the dynamic system in improving sagittal balance and Cobb angles and reducing complications associated with fusion and rigid stabilization procedures, including screw loosening, rod breakage, PJK, and pseudo-arthrosis. The adaptation of deformity classification systems originally designed for rigid systems to dynamic stabilization systems is a promising alternative method for treating patients with ASDs. Further research and surgeon experience are necessary for the long-term viability of dynamic systems in treating degenerative spinal deformities.

## Figures and Tables

**Table 1:** Sacral parameters data.

Silva–Lenke	Number	SS		PT		PI	
		Pre-op	Post-op	Pre-op	Post-op	Pre-op	Post-op
2	56	32.66	32.75	22.13	21.04	52.46	53.39
3	12	33.75	29.00	28.00	29.25	62.00	56.83
Berjano-Lamartine							
1	15	40.93	34.93	17.00	19.33	55.93	54.60
2	18	34.44	33.94	21.28	21.33	55.11	55.28
3	21	30.55	29.73	25.14	24.05	51.59	52.41
4	14	24.43	31.50	31.07	24.43	55.79	54.93

SS: Sacral Slope, PT: Pelvic Tilt, PI: Pelvic Incidence.

**Table 2:** Demographic characteristics of the patients.

Age	62.18 ± 15.42 (49–85)
Women Men	40 (59%) 28 (41%)
Follow-up (months)	39.12 (24–120)

**Table 3:** Surgical outcomes of scoliotic cobb angles according to Berjano–Lamartina and Silva–Lenke classifications.

Silva–Lenke	Scoliotic Cobb Angle		*p*
	Preop (Mean + SD)	Postop (Mean + SD)	
2	13.34 ± 7.77	8.11 ± 4.28	<0.001
3	34.50 ± 3.45	16.50 ± 4.23	<0.001
Berjano-Lamartine			
1	10.47 ± 6.47	5.73 ± 3.31	=0.017
2	16.39 ± 9.49	10.28 ± 4.30	=0.017
3	19.55 ± 12.76	11.05 ± 5.99	=0.083
4	22.21 ± 10.37	11.64 ± 5.47	=0.023

**Table 4:** Variation of patients’ VAS and ODI scores over 24 months.

	Preop	6th Month	12th Month	24th Month	*p* *
	Mean ± SD	Mean ± SD	Mean ± SD	Mean ± SD	
VAS	6.88 ± 1.88 ^a^	2.43 ± 1.11 ^b^	1.21 ± 0.96 ^c^	1.02 ± 0.96 ^c^	<0.001
ODI	68.22 ± 15.34 ^a^	26.95 ± 9.68 ^b^	12.37 ± 10.92 ^c^	10.52 ± 9.07 ^c^	<0.001

* Repeated measure ANOVA analysis was applied. ^a,b,c^: The group from which the difference originates.

## Data Availability

The datasets used and/or analyzed during the current study are available from the corresponding author upon reasonable request.
